# Colony morphology and transcriptome profiling of *Pseudomonas putida* KT2440 and its mutants deficient in alginate or all EPS synthesis under controlled matric potentials

**DOI:** 10.1002/mbo3.180

**Published:** 2014-06-10

**Authors:** Gamze Gulez, Ali Altıntaş, Mustafa Fazli, Arnaud Dechesne, Christopher T Workman, Tim Tolker-Nielsen, Barth F Smets

**Affiliations:** 1 Department of Environmental Engineering, Technical University of DenmarkBuilding 113, Kongens Lyngby, Denmark; 2 Center for Biological Sequencing, Department of Systems Biology, Technical University of DenmarkBuilding 208, Kongens Lyngby, Denmark; 3 Faculty of Science, Department of Biology, University of CopenhagenOle Maaløes Vej 5, Copenhagen, Denmark; 4 Faculty of Health and Medical Sciences, Department of International Health, Immunology, and Microbiology, University of CopenhagenBlegdamsvej 3C, Copenhagen, Denmark

**Keywords:** Bioinformatics, environmental stress, microbial ecology, *Pseudomonas*

## Abstract

*Pseudomonas putida* is a versatile bacterial species adapted to soil and its fluctuations. Like many other species living in soil, *P. putida* often faces water limitation. Alginate, an exopolysaccharide (EPS) produced by *P. putida*, is known to create hydrated environments and alleviate the effect of water limitation. In addition to alginate, *P. putida* is capable of producing cellulose (*bcs*), putida exopolysaccharide a (*pea*), and putida exopolysaccharide b (*peb*). However, unlike alginate, not much is known about their roles under water limitation. Hence, in this study we examined the role of different EPS components under mild water limitation. To create environmentally realistic water limited conditions as observed in soil, we used the Pressurized Porous Surface Model. Our main hypothesis was that under water limitation and in the absence of alginate other exopolysaccharides would be more active to maintain homeostasis. To test our hypothesis, we investigated colony morphologies and whole genome transcriptomes of *P. putida* KT2440 wild type and its mutants deficient in synthesis of either alginate or all known EPS. Overall our results support that alginate is an important exopolysaccharide under water limitation and in the absence of alginate other tolerance mechanisms are activated.

## Introduction

Bacteria living in surface soil and similar porous environments may face fluctuations in the available water due to climatic or anthropological activities. The availability of water is measured as the water potential (Ψ), which expresses the energetic state of water. The major component of the Ψ_m_ under nonsaline conditions is the matric potential (Ψ_m_), which is due to the adsorptive and capillary forces acting on the water in pores (Papendick and Campbell 1981). Ψ_m_, together with the pore geometry, controls the water film thickness (Tuller et al. 1999). As the soil gets drier, the Ψ_m_ becomes more negative and the water film thickness decreases. Depending on how low the Ψ_m_ is, bacteria may experience mild to severe damages and limitations. These can range from dispersal limitation to damage to proteins and nucleic acids, to reduced or ceased growth rate, and even death (Potts 1994; Dechesne et al. 2010). Bacteria can cope with those situations by activating diverse response mechanisms such as accumulating compatible solutes (Potts 1994; Elbein et al. 2003; Fernandez-Aunión et al. 2010), producing universal stress and heat/cold shock proteins (Dos Santos et al., 2004), accumulating fatty acids, and changing fatty acid composition (Kieft et al. 1994; Halverson and Firestone 2000; van de Mortel et al. 2004).

Exopolysaccharide (EPS) production has also long been suspected as a major response to water limitation (Wilkinson 1958). Studies have reported increased EPS production with increased water limitation (Roberson and Firestone 1992) and have described how EPS acts as water-binding agent (Sutherland 2001). The EPS matrix can hold approximately 10 times its weight in water (Roberson and Firestone 1992; Chenu and Roberson 1996). The polymer composition and structure influence the ion-binding and gelation ability of EPS and depending on the type of polysaccharide, the water retention capacity may also change (Or et al. 2007). In addition, it has been suggested that under water-limited conditions, EPS can act as a continuum agent between the soil particles and re-establish hydraulic pathways (Or et al. 2007), which may aid in surface colonization and affect colony morphology.

Nearly all *Pseudomonas* species, including *Pseudomonas putida*, have the ability to produce alginate, a major exopolysaccharide (Halverson, 2009) composed of *β*-1,4 d-mannuronic and l-guluronic acids linked via *β*-1,4-glycosidic bonds (Remminghorst and Rehm 2006). Alginate plays a role in creating hydrated environments (Chang et al. 2007). It helps to alleviate the effect of oxidative stress either by creating a less stressing environment or by acting as antioxidant agent to decrease reactive oxygen species (ROS) in *P. putida* mt-2 (Chang et al. 2009). Moreover, gene expression studies with *Pseudomonas putida* mt-2 under −1.5 MPa Ψ_m_ have revealed upregulation of alginate synthesis genes (van de Mortel and Halverson 2004) and transient expression of alginate genes with respect to biofilm age (Li et al. 2010). Similarly, our previous whole genome microarray study showed a transient expression of alginate synthesis genes of KT2440; at −0.4 MPa Ψ_m_ we found that alginate synthesis genes were upregulated at 4 h of stress but decrease their expression levels with prolonged stress (Gulez et al. 2012). These findings are supporting the role of alginate as an important exopolysaccharide under water limitation.

Alginate, nevertheless, is not the only exopolysaccharide that *P. putida* can produce*. Pseudomonas putida* KT2440 carries the genes for cellulose biosynthesis (Nelson et al. 2002). Moreover, recent studies (Nielsen et al. 2011; Nilsson et al. 2011) confirmed the presence of two other exopolysaccharide gene clusters in *P. putida* KT2440; putida exopolysaccharide a (*pea*) and putida exopolysaccharide b (*peb*), besides alginate (*alg*) and cellulose (*bcs*). Nilsson et al. (2011) evaluated the role of *alg*, *bcs*, *pea*, and *peb* clusters in saturated KT2440 biofilms. They found that *alg*, *bcs*, *pea*, and *peb* knockout mutants could all form biofilms, but the stability of the biofilms formed by the *pea* and *peb* mutants was lower. Hence, they suggested that the products of the *alg* and *bcs* gene clusters are not as important as the products of the *pea* and *peb* gene clusters for biofilm formation. Similar experiments with multiple mutants (mutants that are capable of producing only one of the EPS components: Alg^+^, Bcs^+^, Pea^+^, and Peb^+^) suggested that one EPS gene cluster compensates the absence of other EPS gene clusters (Nilsson et al. 2011). More relevant to our study, Nielsen et al. (2011) investigated the role of *alg*, *bcs*, and *pea* gene clusters of *P. putida* mt-2 under water limiting conditions (at −1.5 MPa Ψ_m_ on PEG 8000 amended solid medium) in addition to biofilm stability and rhizosphere colonization. Based on their gene expression study, they suggested that under water-limiting conditions the products of *pea* and *bcs* genes may contribute to hydration, but not as much as alginate does. Their experiments with multiple mutants also suggested a compensation-like mechanism; in the absence of alginate, Bcs and Pea production can increase and contribute to hydration. Furthermore, they also found that Bcs and alginate, but not Pea, contribute to rhizosphere colonization.

Although Nielsen et al. (2011) provided valuable information; they simulated the Ψ_m_ using PEG 8000 additions, this method does not account for the water film thickness effect as experienced in porous environments. Here, we used the Pressurized Porous Surface Model (PPSM) to grow *P. putida* under directly controlled matric potentials resulting in the creation of thin water films (Gulez et al. 2010). Moreover, since Nielsen et al. (2011) did not examine whole genome transcriptome, their study does not inform on how the absence of EPS synthesis genes affects the expression of the other genes in *P. putida* under water limitation. Hence, we examined colony morphologies and whole genome transcriptome profiles of the WT and Alg^−^ under water-limited (−0.4 MPa Ψ_m_) and water-replete (−0.5 kPa Ψ_m_) conditions. Our main hypothesis was that under water limitation and in the absence of alginate synthesis genes other exopolysaccharides would be more active to maintain homeostasis in *P. putida*. More specifically we hypothesized that under water limitation (−0.4 MPa Ψ_m_), colony morphology of the Alg^−^ would be similar to the WT's morphology and genes belonging to either of the exopolysaccharide gene clusters (*pea*, *peb*, and *bcs*) would be upregulated in the Alg^−^ mutant. A second mutant deficient in complete EPS synthesis (EPS^−^) was also included in our experiments to analyze colony morphologies and identify alternative responses.

## Materials and Methods

### Bacterial strains

*Pseudomonas putida* KT2440 wild-type (WT) and two of its mutants deficient either in alginate (Alg^−^) or all known EPS (EPS^−^) synthesis were used in this study (deleted genes in Alg^−^: PP1277-PP1288; in EPS^−^: PP1277-1288 [*alg*] + PP2634-2638 [*bcs*] + PP3132-3142 [*pea*] + PP1795-1788 [*peb*]) (Nilsson et al. 2011). Depending on the aim of the experiment both *gfp*-tagged and -untagged strains were used.

### Experimental platform

For the experiments under defined Ψ_m_ we used the Porous Surface Model (PSM) and PPSM as explained in Dechesne et al. (2008) and Gulez et al. (2010). Briefly, both PSM and PPSM consist of a filter holder (BONTEC-AS, Ballerup, Denmark) in which a porous ceramic plate (7.1-mm thick and 41.3 mm in diameter, 5-bar bubbling pressure plate; Soilmoisture, Santa Barbara, CA) is located. A silicone O-ring (40 mm in inner diameter, 5-mm thick) that surrounds the plate makes the system airtight. The filter holder is connected to a reservoir of growth medium through a silicone tubing. In the PSM, the Ψ_m_ is set by changing the hydraulic head between the surface of the ceramic plate and that of the air–liquid interface in the medium reservoirs (Dechesne et al. 2008); in the PPSM, the Ψ_m_ is set by applying positive pressure using compressed gas (8% O_2_ in N_2_), required to create −0.4 MPa matric stress (Gulez et al. 2010). Prior to inoculation, the systems were autoclaved at 121°C for 25 min.

### Colony growth and morphology analysis

Cultures of *P. putida* KT2440 WT, Alg^−^, and EPS^−^ were grown overnight on LB plates and suspended in 0.9% NaCl prior to inoculation. On the (P)PSM, the strains were grown on ABTB medium with benzoate as the carbon source (1 mmol/L MgCl_2_, 0.1 mmol/L CaCl_2_, 0.01 mmol/L Fe-EDTA, 15 mmol/L (NH_4_)_2_SO_4_, 33 mmol/L Na_2_HPO_4_, 22 mmol/L KH_2_PO_4_, 51 mmol/L NaCl, and 20 mmol/L benzoate). Two microliters of cell suspension (approximately 1 × 10^5^ cells/*μ*L) were pipetted on top of the ceramic plates (three plate replicates for each strain and Ψ_m_) and incubated for 5 days at either −0.4 MPa (PPSM set-up, water-limited condition) or −0.5 kPa Ψ_m_ (PSM set-up, water-replete condition). At the end of the incubation period, units were disassembled, and images of up to nine colonies per filter were captured using Leica MZ16 FA epifluorescent stereomicroscope. Colony morphologies were characterized by measuring shape parameters (area, perimeter, roundness, fractal dimension) (Cenens et al. 2002) using Image Pro Plus software (version 5; Media Cybernetics, Silver Spring, MD) and ImageJ (Rasband, –2012^1997^).

### Incubations for microarray analysis

Aliquots of overnight grown *P. putida* KT2440 wild type and mutant strains were inoculated (approximately 1 × 10^7^ cells in 100 *μ*L) on the surface of ceramic plates. Total duration of the incubation was always 5 days, after which the cells were harvested (see below). The cells were either maintained at −0.5 kPa (water-replete condition) until the termination of experiment (control) or subjected to −0.4 MPa ψ_m_ (water-limited condition) for the last 4 h of the 5-day incubation period. Four replicate PPSMs were incubated for each condition and each strain.

### Sampling, RNA extraction, RT, and labeling

At the end of the incubation period the (P)PSMs were quickly dissembled and the cells were flooded with stop solution (5% phenol in 100% ethanol) (Cytryn et al. 2007), and harvested with a cytological brush (Gynobrush, Heinz Herenz, Hamburg, Germany). Harvested cells were suspended in 2-mL Eppendorf tubes containing 200 mL of the stop solution. Immediately after, RNA extraction was performed using Agilent MiniRNA Kit (Palo Alto, CA) following the manufacturer's protocol. RNA concentrations and integrities were checked using NanoDrop 1000 (Thermo Fisher Scientific, Rockland, DE), Qubit 2.0 Fluorometer (Invitrogen, Carlsbad, CA), Agilent 2100 Bioanalzyer (Palo Alto, CA), and gel electrophoresis. Labeling and cDNA synthesis were performed as per manufacturer's protocol (One-Color Microarray-Based Gene Expression Analysis Low Input Quick Amp Labeling; Agilent).

### Microarray and hybridization conditions

A custom-made Nimblegen (Madison, WI) whole genome one-color oligonucleotide expression array (12 × 135 K with 45–60 mer probes) of *P. putida* KT2440 was used as the microarray platform. Labeled cDNA was hybridized to the array probes on the array as per manufacturer's protocol (Nimblegen Hybridization System). After hybridization, spot intensities were acquired by scanning the arrays (GenePix 4000B; Molecular Devices, Sunnyvale, CA).

### Microarray data analysis

Gene expression data were analyzed with the statistical software R (http://www.r-project.org) using the *oligo* and *limma* packages available in Bioconductor (http://www.bioconductor.org). Data were normalized using Robust Multiarray Average (RMA) approach with the quantile method. Differentially expressed genes were determined by comparing expression values of each mutant relative to the WT for each Ψ_m_ (Comparison I) and also under −0.4 MPa Ψ_m_ relative to values at −0.5 kPa Ψ_m_ for each strain (Comparison II). Those genes with average absolute log_2_-fold change equal or larger than 1.5 and false discovery rate (FDR) <0.01 were identified as significantly differentially expressed genes. Finally, gene annotations were made using the annotation files available in the Comprehensive Microbial Resource (CMR) website (http://cmr.jcvi.org/). Categorization of significant genes based on their roles and subroles were made using CMR website.

### Protein–protein interaction network analysis

Protein–protein interaction network analysis was performed using Cytoscape software (Cline et al. 2007). The protein–protein interaction network is acquired from Park et al. (2009). Protein–protein interactions regarding alginate genes were added manually to the network based on the literature (Wozniak and Ohman 1994; Remminghorst and Rehm 2006). We extracted the networks that are only composed of significantly differentially expressed genes in either of the strains. The subnetworks were generated by selecting the first neighbors. Confidence scores for each interaction (Park et al. 2009) are listed in Table S3.

### qRT-PCR

To partially validate the trends of the microarray results, we performed qRT-PCR on the isolated RNA for selected genes with three replicates for each condition and strain (WT, Alg^−^, and EPS^−^ under −0.4 MPa and −0.5 kPa). The genes and their primer sets are listed in Table S4. cDNA synthesis and amplification were performed using Qscript 1-Step SYBR Green QRT-PCR Kit (Quanta Biosciences, Gaithersburg, MD) in a Chromo4 thermocycler (MJ Research, Waltham, MA) with a total RNA input of 50 ng following the standard protocol as per the manufacturer (Quanta Biosciences). Data were normalized with respect to *rimM* (coding for the 16S rRNA processing protein RimM), which was not differentially expressed in our study, as done by Li et al. (2010) and Gulez et al. (2012). Expression levels of the target genes at −0.4 MPa were calculated relative to levels at −0.5 kPa using the 2^▵▵CT^ method (Livak and Schmittgen 2001).

### Accession to the microarray data

The microarray data can be accessed through GEO database with the accession number GSE53407 (http://www.ncbi.nlm.nih.gov/geo/query/acc.cgi?acc=GSE53407).

## Results and Discussion

### Colony growth and morphology

**Figure 1:**
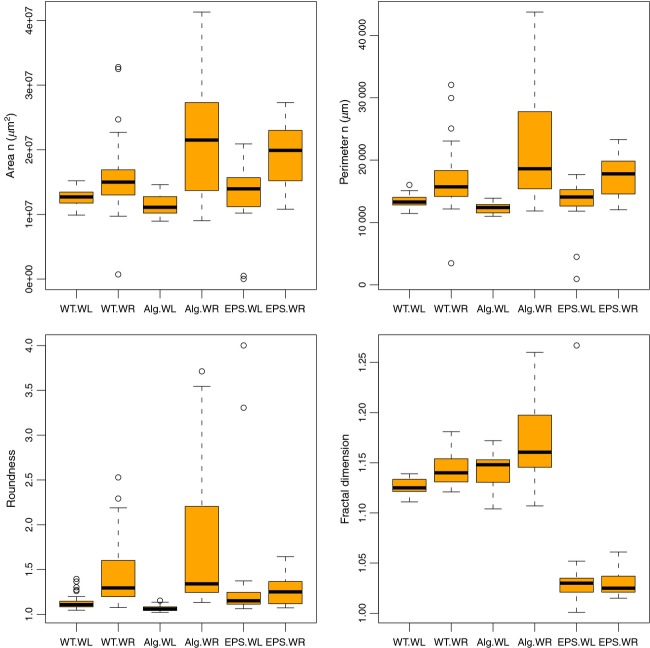
Box plots of the morphological parameters in the colonies of *Pseudomonas putida* KT2440 WT, alginate mutant (Alg^−^), and EPS mutant (EPS^−^) under water-limitation (WL) and water-replete (WR) conditions. The median of each dataset is indicated by the black line within the box. The edges of rectangles correspond to the first and third quartile of the data set. The open dots represent the outliers. EPS, exopolysaccharide.

To evaluate how EPS deficiency and water limitation affect colony growth, we compared the area, perimeter, roundness, and fractal dimension values of the WT, Alg^−^, and EPS^−^ colonies. As the Box plot shows (Fig.1), under −0.4 MPa Ψ_m_, colonies of all three strains have similar median values and data variation for the area, perimeter, and roundness. This suggests that under −0.4 MPa Ψ_m_, absence of alginate or all the exopolysaccharides does not affect the colony morphologies in terms of the measured parameters. Under −0.5 kPa Ψ_m_, however, WT colonies have lower median values for the area, perimeter, and roundness compared to mutant colonies. The absence of EPS genes might have lowered the metabolic burden associated with carrying those genes and their respective functions so that cells could invest in colony growth and dispersal (Nadell and Bassler 2011). Figure1 also shows that under −0.5 kPa Ψ_m_ conditions data variation was much higher for all the strains, being highest for the Alg^−^.

In addition to abovementioned parameters, we calculated fractal dimension values (*D*_f_) of the colonies for each strain. *D*_f_ gives information about the irregularity of the edges of a colony. A *D*_f_ value of 1 indicates smooth edges, whereas values >1 indicate more irregularities at the colony edges. WT and Alg^−^ colonies had more irregular colony edges compared to the EPS^−^ colonies, regardless of the Ψ_m_. This suggests that other exopolysaccharides may play a role in maintaining irregular colony edges. As for the EPS^−^, having smoother colony edges is probably related to the deletion of all the exopolysaccharide gene clusters. However, since *D*_f_ values were similar under −0.4 MPa Ψ_m_ and −0.5 kPa Ψ_m_, this behavior is again not related to water limitation.

Overall our results indicated that under −0.4 MPa Ψ_m_ there was no substantial difference between the WT and the mutants in terms of colony growth and morphologies. Considering that Chang et al. (2007) observed significant EPS production at −1.5 MPa (∼30.2 *μ*g of uronic acid per mg of protein) but only little at −0.5 MPa (∼6 *μ*g of uronic acid per mg of protein), it is possible that the −0.4 MPa Ψ_m_ created in our study may not have resulted in substantial EPS production. Indeed our attempts to quantify EPS production were inconclusive because of the low-level EPS and high data variation (data not shown). However, since the role of EPS under mild water limitation has already been established (Chang et al. 2007; Li et al. 2010) and since our previous study indicated expression of alginate genes at −0.4 MPa (Gulez et al. 2012), we expected to see transcript level differences between the WT and mutants. Therefore in the next step, we profiled expression of the whole genome of the WT and EPS mutants.

### Gene expression profiling: comparison I

In comparison I, we evaluated how the mutations affected the gene expression profiles. To do that, we detected significantly differentially expressed genes of each mutant relative to the WT under −0.4 MPa Ψ_m_ and −0.5 kPa ψ_m_. Table S1 shows the complete list of the significant genes with their role categories.

As shown in Table1, under −0.4 MPa Ψ_m_ more genes were differentially expressed compared to the −0.5 kPa Ψ_m_. The highest number of expressed genes was observed in the case of Alg^−^ vs. WT. The most important finding is the upregulation of the genes in *peb* cluster, suggesting that absence of alginate may be compensated by upregulating the *peb* genes. Other identified overexpressed genes play roles in energy metabolism, adaptation to atypical conditions, and regulatory functions. A number of hypothetical proteins also take attention with their higher expression levels. In the case of EPS^−^ versus WT, genes belonging to similar role categories were differentially expressed, but not as many as in the Alg^−^ versus WT case.

**Table 1:** Number of significantly differentially expressed genes when comparing the alginate (Alg^−^) and all EPS-deficient (EPS^−^) mutants to the wild type (WT) or to each other under water-limited (−0.4 MPa ψ_m_) and water-replete (−0.5 kPa ψ_m_) conditions (Comparison I)

Compared strains of *Pseudomonas putida* KT2440	−0.4 MPa (dry)	−0.5 kPa (wet)
Alg^−^ vs. WT	34	5
EPS^−^ vs. WT	9	6
Alg^−^ vs. EPS^−^	2	0

Under −0.5 kPa Ψ_m_, regardless of the strain, the majority of the expressed genes were iron-binding proteins (Table S1). Iron-binding proteins are important for iron acquisition and iron is essential for biofilm formation in *P. aeruginosa* (Banin et al. 2005). Iron also influences the surface motility of *P. putida* KT2440 (Matilla et al. 2007). EPS is known to bind metals in *Pseudomonas* and other species and absence of EPS results in lower metal uptake (Lau et al. 2005; Ueshima et al. 2008). It is possible that absence of EPS genes resulted in the downregulation of the iron-uptake mechanisms. Some of these genes were also expressed under −0.4 MPa Ψ_m_, suggesting that these are not water-limitation-specific responses, but rather mutation specific.

Finally, we detected differentially expressed genes in Alg^−^ versus EPS^−^. Under −0.4 MPa Ψ_m_, there were only two significantly expressed genes. Both of those genes were hypothetical with unknown functions. Under −0.5 kPa Ψ_m_, no single gene was significantly differentially expressed. This suggests that mutants behave similarly regardless of the Ψ_m_ they were exposed to in our study.

### Gene expression profiling: comparison II

**Figure 2:**
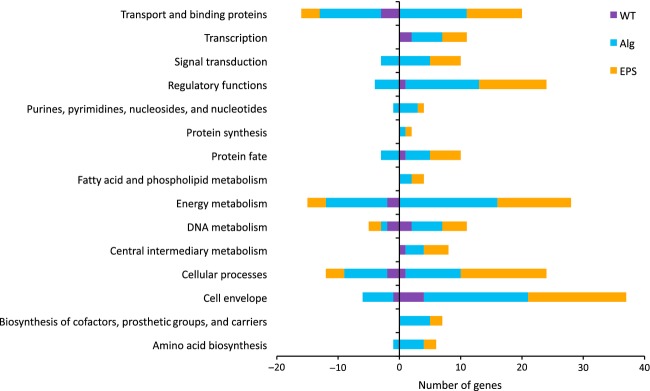
Number of up- and downregulated genes in *Pseudomonas putida* KT2440 WT strain and its mutant strains deficient in alginate (Alg^−^) or all EPS (EPS^−^) synthesis under water-limited (−0.4 MPa Ψ_m_) relative to water-replete (−0.5 kPa Ψ_m_) conditions. Data are grouped according to the major role categories. Positive and negative numbers correspond to the up- and downregulated genes, respectively.

Here, we evaluated how mild water limitation affected the gene expression profiles. Hence, we detected significantly differentially expressed genes under −0.4 MPa Ψ_m_ relative to −0.5 kPa ψ_m_ for each strain. We detected 37, 235, and 169 significantly differentially expressed genes in WT, Alg^−^, and EPS^−^ strains, respectively. The complete list of differentially expressed genes with their subcategories and fold changes are presented in Table S2. The high number of genes expressed in both Alg^−^ and EPS^−^ indicates that EPS mutants respond to mild water limitation more dramatically than WT. Although one may expect to detect more differentially expressed genes in EPS^−^ compared to Alg^−^, our results indicated the opposite. However, as Figure2 shows, most of the genes that were expressed in both mutants belongs to the same role categories, suggesting that cells need to activate similar genes to carry on their basic activities. It is possible that deletion of four different operons in EPS^−^ could affect the functioning of the other stress-related genes and to cope with such a loss in EPS^−^ does not activate as many genes as in Alg^−^.

#### qRT-PCR validation of the microarray data

Using qRT-PCR we partially validated the expression of selected genes (Fig. S1). For the genes tested, qRT-PCR mostly validated the trend for EPS^−^. Both in microarray and qRT-PCR most of the EPS^−^ genes had high fold change values compared to the WT and Alg^−^ (Fig. S1). For the Alg^−^ qRT-PCR produced more or less similar fold-change values for half of the genes tested, whereas the other half mostly resulted in a bit weaker signals than in microarray. For the WT, except for a good correlation for the *algT* gene, qRT-PCR mostly detected stronger signals compared to the microarray. Although it is customary to use qRT-PCR for validating microarray results, the results may not always be in agreement (Chuaqui et al. 2002; Morey et al. 2006). Many factors from PCR biases in qRT-PCR to the application of different data analysis methods could affect the outcome. However, the general trend, in the gene expression values of the EPS^−^ and Alg^−^ genes, with a few exception, was conserved both in microarray and qRT-PCR.

#### EPS synthesis genes and sigma factors

Most of the alginate biosynthesis genes were highly expressed in the WT (Table2). No other exopolysaccharide gene was differentially expressed, except for PP_3138 in the Pea operon which was downregulated. In contrast, Nielsen et al. (2011) observed upregulation of *pea* and *bcs* genes, although not to the extent of alginate genes. This could be related to their way of controlling the water potential. Yet, despite of some differences in our methods and outcomes, alginate still seems to be the primary exopolysaccharide under −0.4 MPa Ψ_m_, supporting the previous studies.

**Table 2:** Log_2_-fold changes for exopolysaccharide synthesis related genes in *Pseudomonas putida* KT2440 WT and its mutants deficient in EPS synthesis when comparing cells grown under water-limited (−0.4 MPa Ψ_m_) relative to water-replete (−0.5 kPa Ψ_m_) conditions (Comparison-II)

Operon	Gene ID	Gene name	Gene annotation	WT	Alg^−^	EPS^−^
	PP_0133	*algB*	Alginate biosynthesis transcriptional regulatory protein AlgB	0.52	2.59	2.70
algT operon	PP_1427	*algT*	RNA polymerase sigma-H factor AlgT	1.68	1.84	2.78
	PP_1428	*mucA*	Sigma factor algU negative regulatory protein MucA	1.66	2.29	2.66
	PP_1429	*mucB*	Sigma factor algU regulatory protein MucB	1.76	2.63	2.72
AlgD operon	PP_1277	*algA*	Mannose-1-phosphate guanylyltransferase/mannose-6-phosphate isomerase	2.89	N/A	N/A
	PP_1278	*algF*	Alginate O-acetyltransferase	3.16	N/A	N/A
	PP_1279	*algJ*	Alginate O-acetylation protein AlgJ	0.87	N/A	N/A
	PP_1280	*algI*	Alginate O-acetylation protein AlgI	0.52	N/A	N/A
	PP_1281	*algL*	Alginate lyase	0.48	N/A	N/A
	PP_1282	*algX*	Alginate biosynthesis protein AlgX	0.45	N/A	N/A
	PP_1283		alginate-C5-mannuronan-epimerase AlgG, putative	0.44	N/A	N/A
	PP_1284	*algE*	Outer membrane protein AlgE	0.72	N/A	N/A
	PP_1285	*algK*	Alginate biosynthesis regulator AlgK	0.83	N/A	N/A
	PP_1286		Alginate biosynthesis protein Alg44	0.21	N/A	N/A
	PP_1287		Alginate biosynthesis protein Alg8	0.49	N/A	N/A
	PP_1288	*algD*	GDP-mannose 6-dehydrogenase	3.26	N/A	N/A
Bcs operon	PP_2634		Cellulose synthase, putative	0.10	−0.03	N/A
	PP_2635		Cellulose synthase, putative	0.13	0.08	N/A
	PP_2636		Cellulose synthase, putative	−0.01	−0.01	N/A
	PP_2637		Endo-1,4-beta-d-glucanase	0.21	0.02	N/A
	PP_2638		Cellulose synthase operon C protein, putative	−0.02	−0.10	N/A
Pea operon	PP_3132		Polysaccharide transporter, putative	−1.29	−1.79	N/A
	PP_3133		Oxidoreductase, putative	−0.34	−0.53	N/A
	PP_3134		Conserved domain protein	−1.87	−2.65	N/A
	PP_3135		Glycosyl transferase, putative	−1.42	−2.22	N/A
	PP_3136		Serine O-acetyltransferase, putative	−0.71	−1.01	N/A
	PP_3137		Glycosyl transferase, group 2 family protein	−0.42	−0.69	N/A
	PP_3138		VirK domain protein	−1.65	−1.78	N/A
	PP_3139		Glycosyl transferase, group 1 family protein	−0.41	−0.56	N/A
	PP_3140		Glycosyl transferase, group 2 family protein	−0.26	−0.09	N/A
	PP_3141		Glycosyl transferase, WecB/TagA/CpsF family	−0.04	−0.40	N/A
	PP_3142		Sugar transferase, putative	−0.96	−0.80	N/A
Peb operon	PP_1788		Hypothetical protein	−0.21	2.29	N/A
	PP_1789		Hydrolase, haloacid dehalogenase-like family	0.05	2.10	N/A
	PP_1790		Acylneuraminate cytidylyltransferase, putative	−0.39	1.01	N/A
	PP_1791		Aldolase/synthase, putative	−0.12	1.01	N/A
	PP_1792		Glycosyl transferase, group 2 family protein	−0.09	1.29	N/A
	PP_1793		Glycosyl transferase, group 2 family protein	−0.13	0.91	N/A
	PP_1794		Hypothetical protein	−0.91	0.82	N/A
	PP_1795		Hypothetical protein	0.25	0.91	N/A

Shaded cells and “N/A” indicate nonsignificant and deleted genes, respectively. Only genes with both an absolute log_2_-fold change equal or larger than 1.5 and false discovery rate (FDR) <0.01 are labeled as significantly expressed.

**Figure 3:**
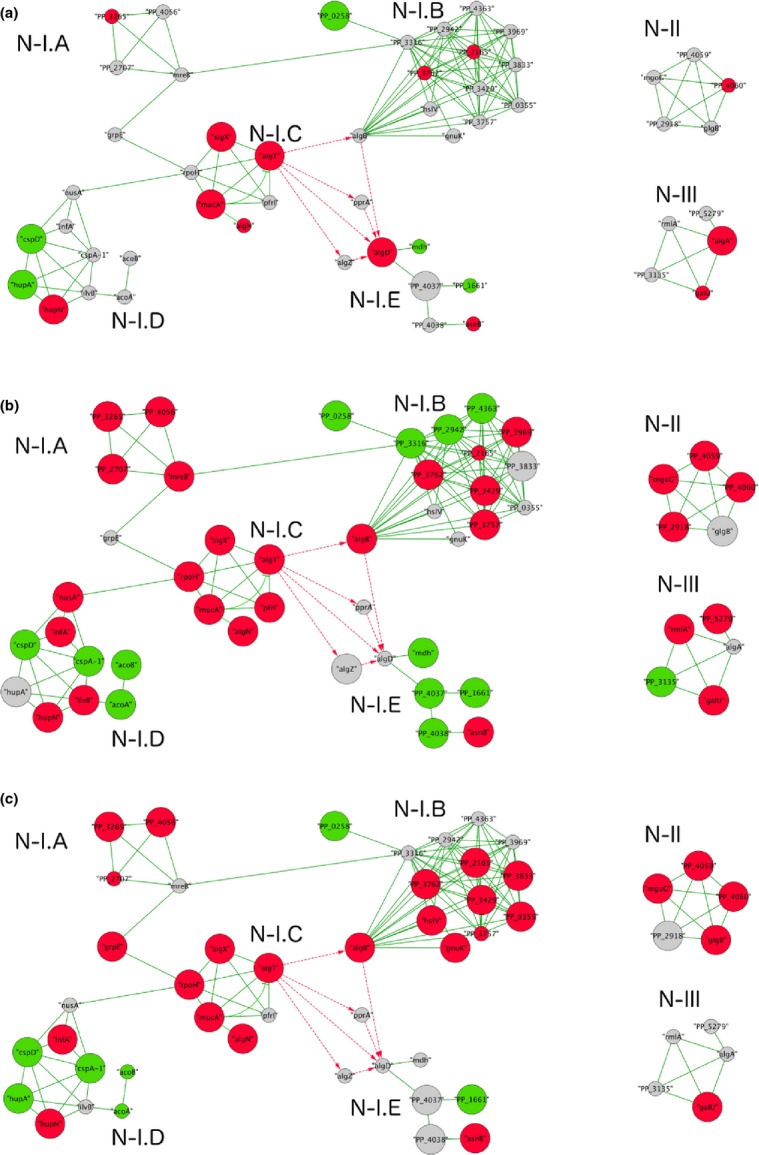
Protein–protein interaction networks showing significantly differentially expressed genes in *Pseudomonas putida* KT2440 strains under water-limited (−0.4 MPa Ψ_m_) relative to water-replete (−0.5 kPa Ψ_m_) conditions: (A) wild type, (B) alginate mutant, (C) EPS mutant. Node sizes correspond to statistical significance based on FDR with a bigger node corresponding to higher FDR. Red, green, and gray nodes correspond to upregulated, downregulated, and nonsignificant genes, respectively. Edge colors correspond to the interactions retrieved by Park et al. (2009) as green and by Wozniak and Ohman (1994) and Remminghorst and Rehm (2006) as dotted red. A high-resolution version of the network is available as supplementary file (Fig. S2).

Looking at the mutants, we see that Alg^−^ upregulated some of the genes in Peb operon and downregulated a few genes in the Pea operon (Table2). Expression of some *peb* genes did not seem to substantially compensate the absence of alginate synthesis genes as the number of expressed genes was quite high in the Alg^−^ strain. We also mapped the significantly differentially expressed genes onto the predicted protein–protein interaction network of *P. putida* KT2440 acquired from Park et al. (2009). From that network we extracted the subnetworks that were only formed by the significantly differentially expressed genes in either of the strains we studied (Fig.3). The protein–protein interaction network revealed a network of exopolysaccharide-related proteins; some involved in alginate, some in Pea synthesis (Fig.3 N-III). Since *algA* was deleted in the mutants, we naturally did not see expression of this gene in the Alg^−^ and EPS^−^. However, *algA* deletion seems to activate many genes in this network for the Alg^−^ strain including *rmlA* and *galU*. The Alg^−^ strain also displayed downregulation of PP_3135, which is a gene in the Pea operon. In EPS^−^ strain no genes except *galU* were upregulated. The protein–protein interaction network also revealed another cluster, which is partly composed of alginate biosynthesis proteins (Fig.3N-I.E) including AlgD. *algD* is the first gene in the alginate synthesis operon. In WT, *algD* was significantly differentially expressed under −0.4 MPa Ψ_m_. Some of these genes were absent in the mutants, however, the only gene that was upregulated in the mutants was *asnB* gene, annotated as asparagine synthetase.

Interestingly, alginate synthesis regulatory genes (*algB*, *algT*, *mucA*, *mucB*) were also overexpressed in both mutants; with the expression levels highest for EPS^−^. In *P. aeruginosa*, *algT*, *mucA*, and *mucB* are suggested to be the top genes responsible to determine the alginate-producing phenotype (Wozniak and Ohman 1994). MucA inhibits AlgT activity; thereby inhibiting alginate synthesis in *P. aeruginosa* and MucB might have a role balancing the activity between AlgT and MucA (Xie et al. 1996). In *P. aeruginosa*, AlgT is required for *algB* transcription (Wozniak and Ohman 1994) and AlgB is required for *algD* transcription, which is the first gene in the alginate synthesis operon. The fact that all the genes upstream of alginate biosynthesis are upregulated, especially *algB*, suggests that there may not be a feedback to stop the alginate operon to be transcribed. Alternatively, these genes may also control other functions than alginate biosynthesis.

Indeed Figure3N-1.C shows that AlgT interacts with many alternative sigma factors, such as RpoH, SigX, and PfrI, which are associated with stress tolerance. Figure3 shows that the WT only upregulated *algT*, *sigX*, and *mucA*, whereas the Alg^−^ upregulated all the genes in this network and the EPS^−^ upregulated all but *pfrI*. As sigma factors are important under environmental stress, it is likely that absence of exopolysaccharide genes resulted in the activation of more sigma factors to generate alternative responses. In *P. aeruginosa*, AlgT is known to control the expression of *rpoH* (*σ*^32^) (Schurr and Deretic 1997). RpoH sigma factor, controls the heat shock regulon in *Escherichia coli* (Grossman et al. 1987). The role of RpoH in *P. putida* is not fully understood; there is evidence that it is upregulated under heat shock (Aramaki et al. 2001), although other studies could not identify this function (Manzanera et al. 2001). Our results indicated significant upregulation of *rpoH* in both Alg^−^ and EPS^−^ suggesting that RpoH plays a role in water limitation.

The sigma factor SigX was expressed in all the strains. It is known to control the transcription of *oprF* gene, the product of which is an outer membrane protein (Brinkman et al. 1999). However, in our study we did not detect significant *oprF* expression. The sigma factor PfrI is only upregulated in Alg^−^. It is known to play a role in iron uptake (Leoni et al. 2002). It is possible that in the absence of alginate, iron may not be retained in the cell's microenvironment and KT2440 activates iron-scavenging mechanism. However, this does not explain why in EPS^−^ the same gene was not expressed.

#### Oxidative stress-related genes

A secondary effect of water limitation is the accumulation of ROS, which causes oxidative stress (Potts et al. 2005; Garcia 2011). ROS can damage DNA and proteins and also have a toxic effect on the cells. Therefore, quite a few mechanisms are dedicated to cope with oxidative stress, either by activating detox mechanisms or antioxidant production (Mailloux et al. 2009; Booth et al. 2011). The oxidative stress response genes are involved in the TCA cycle, glyoxylate cycle, amino acid synthesis, and glutathione synthesis. We detected significant expression of these genes in all the strains. The number and expression levels of the genes were lower in the WT compared to the mutants. Knowing that alginate alleviates the effect of oxidative stress (Chang et al. 2009), it is no surprise that in the absence of exopolysaccharides, genes involved in oxidative stress tolerance were highly expressed.

#### Membrane integrity-related genes

In the cell envelope, especially the outer membrane part of it, environmental stress is first sensed and potential damage (denaturation, fluidity change, etc.) may occur. Exopolysaccharide production is one way to maintain the membrane integrity and function by alleviating the water stress. There are, however, other mechanisms by which the damage is controlled (Potts 1994).

One important way to prevent denaturation of cell membrane and proteins is the accumulation of compatible solutes (Potts 1994; Elbein et al. 2003). Among many compatible solutes, trehalose has been the center of attention for many species, especially under extreme water deprivation (Potts 1994). Mannitol, *N*-acetylglutaminylglutamine amide (NAGGN), and betaine are listed as other compatible solutes besides trehalose, which *P. putida* can produce (Kets et al. 1996a,b). We did not detect significant expression of any compatible solute related gene in the WT. As for the mutants, we did not detect any betaine gene in any of the strains. However, both mutants expressed PP1749 and PP1750 genes, which are probably involved in NAGGN synthesis like their ortholog genes PA3459 and PA3460 in *P. aeruginosa* (Aspedon et al. 2006). Moreover, the Alg^−^ and EPS^−^ strains expressed a few genes responsible for trehalose synthesis (PP2918 was only expressed in Alg^−^, PP4050 (*glgA*) and 4058 (*glgB*) are only expressed in EPS^−^, and PP 4059 is expressed both mutants). Our network analysis also revealed a network composed of proteins that are involved in trehalose synthesis (Fig.3N-II). While the WT did not have any significantly expressed genes in this network, the Alg^−^ and EPS^−^ expressed all the genes (even the nonsignificantly expressed genes have values closer to be classified as significant genes). This suggests that when exopolysaccharides genes are absent, especially the alginate genes, trehalose genes are upregulated to compensate the absence as stated before.

Under water stress, membrane fluidity changes and one way to maintain it is to change the fatty acid composition; either by changing the relative amount of *cis*–*trans* isomers or saturated–unsaturated fatty acids (Halverson and Firestone 2000; van de Mortel et al. 2004). Fatty acid synthesis genes, outer membrane lipoproteins, and cell envelope-related genes were expressed in the mutants (listed under the “cell envelope/other” and “fatty acid and phospholipid metabolism” categories in Table S2) indicating possible changes at membrane fluidity in response to mild water limitation. Our results indicate that in the absence of EPS these mechanisms become more important in generating stress tolerance responses.

#### Other genes

Genes involved in protein fate (heat shock proteins, universal stress proteins) were also expressed in all the strains. However, EPS mutants expressed more of those genes than the WT. Network I.D (Fig.3) is composed of stress response proteins ranging from cold shock proteins to dehydrogenases, which play a role in adaptation to atypical conditions. As this network shows, the number of significantly expressed genes was lowest for the WT, *hupN* is upregulated and *hupA* and *cspD* are downregulated. HupN and CspD have the same trends in Alg^−^ and EPS^−^. *NusA* is highly expressed in Alg^−^, but not in the other strains. The network analysis also revealed a network of interest, which is composed of hypothetical proteins and DNA replication/repair proteins (Fig.3N-1.A). Under water limitation the WT did not express any genes in Network I.A, whereas the Alg^−^ and EPS^−^ expressed a majority of these genes.

Except *mucA*, no genes in the regulatory functions and signal transduction were expressed in the WT. However, many genes within the regulatory function category were highly expressed in the mutants. Our network analysis also showed a cluster in Network I, which contains response regulator and signaling proteins (Fig.3N-1.B). As shown in the Figure3, in the WT none of the genes were significantly expressed, except the downregulation of the PP_0258. However, many genes including *algB* were expressed in the Alg^−^ and EPS^−^. As this network shows that AlgB interacts with many other proteins involving in response regulation, it is possible that AlgB may be involved in controlling other functions in KT2440.

To summarize, our study showed that absence of exopolysaccharides did not have a significant effect on the colony morphology under −0.4 MPa Ψ_m_. Our microarray study, on the hand, revealed that absence of exopolysaccharide synthesis genes, mainly alginate, had a dramatic effect at the gene expression level under −0.4 MPa ψ_m_. Besides, the microarray results also supported some of the predicted protein–protein interactions (Park et al. 2009) as shown in Figure3. The high number of expressed genes and their higher expression levels in the exopolysaccharide mutants supports the previous findings about alginate being an important exopolysaccharide in *P. putida* under mild water limitation. Furthermore, expression of some alginate regulatory genes (*algT*, *algB*) and many genes belonging to other stress tolerance responses in the mutants suggests that those regulatory genes may also be involved in controlling other cellular activities under mild water limitation. However, the exact role of alginate and the regulatory genes should be investigated in a follow-up study.

## Supporting Information

Figure S1 qRT-PCR quantification of the selected transcripts in *Pseudomonas putida* KT2440 WT and its mutants deficient in exopolysaccharide synthesis (Alg^−^ and EPS^−^) under water-limited (−0.4 MPa Ψ_m_) relative to water-replete (−0.5 kPa Ψ_m_) conditions. Error bars stand for the standard deviations of the fold-change values.

Figure S2 Protein–protein interaction network showing significantly differentially expressed genes in *Pseudomonas putida* KT2440 strains under water-limited (−0.4 MPa Ψ_m_) relative to water-replete (−0.5 kPa Ψ_m_) condition: (A) WT, (B) alginate mutant, (C) EPS mutant. Node size corresponds to statistical significance based on FDR where a bigger node corresponds to higher FDR. Red, green, and gray nodes correspond to upregulated, downregulated, and nonsignificant genes, respectively. Edge colors correspond to the interactions retrieved by Park et al. (2009) as green and by Wozniak and Ohman (1994) and Remminghorst and Rehm (2006) as dotted red.

Table S1 Log_2_-fold changes for significantly differentially expressed genes when comparing the *Pseudomonas putida* KT2440 EPS-deficient mutant cells to the WT cells or to each other under water-limited (−0.4 MPa Ψ_m_) and water-replete (−0.5 kPa Ψ_m_) conditions (Comparison I)

Table S2 Log_2_-fold changes for significantly differentially expressed genes listed according to their role categories in *Pseudomonas putida* KT2440 WT and its mutants deficient in EPS synthesis when comparing cells grown under water-limited (−0.4 MPa Ψ_m_) relative to water-replete (−0.5 kPa Ψ_m_) conditions (Comparison II).

Table S3 Confidence scores of the interactions in the protein–protein interaction network of *Pseudomonas putida* KT2440 acquired by Park et al. (2009)

Table S4 Primers used in the qRT-PCR
