# Supplementary figures and images for: Colony morphology and transcriptome profiling of Pseudomonas putida KT2440 and its mutants deficient in alginate or all EPS synthesis under controlled matric potentials
Source: Microbiologyopen. 2014 Jun 10;3(4):457–69. doi: 10.1002/mbo3.180 (PMC4287175; doi:10.1002/mbo3.180)

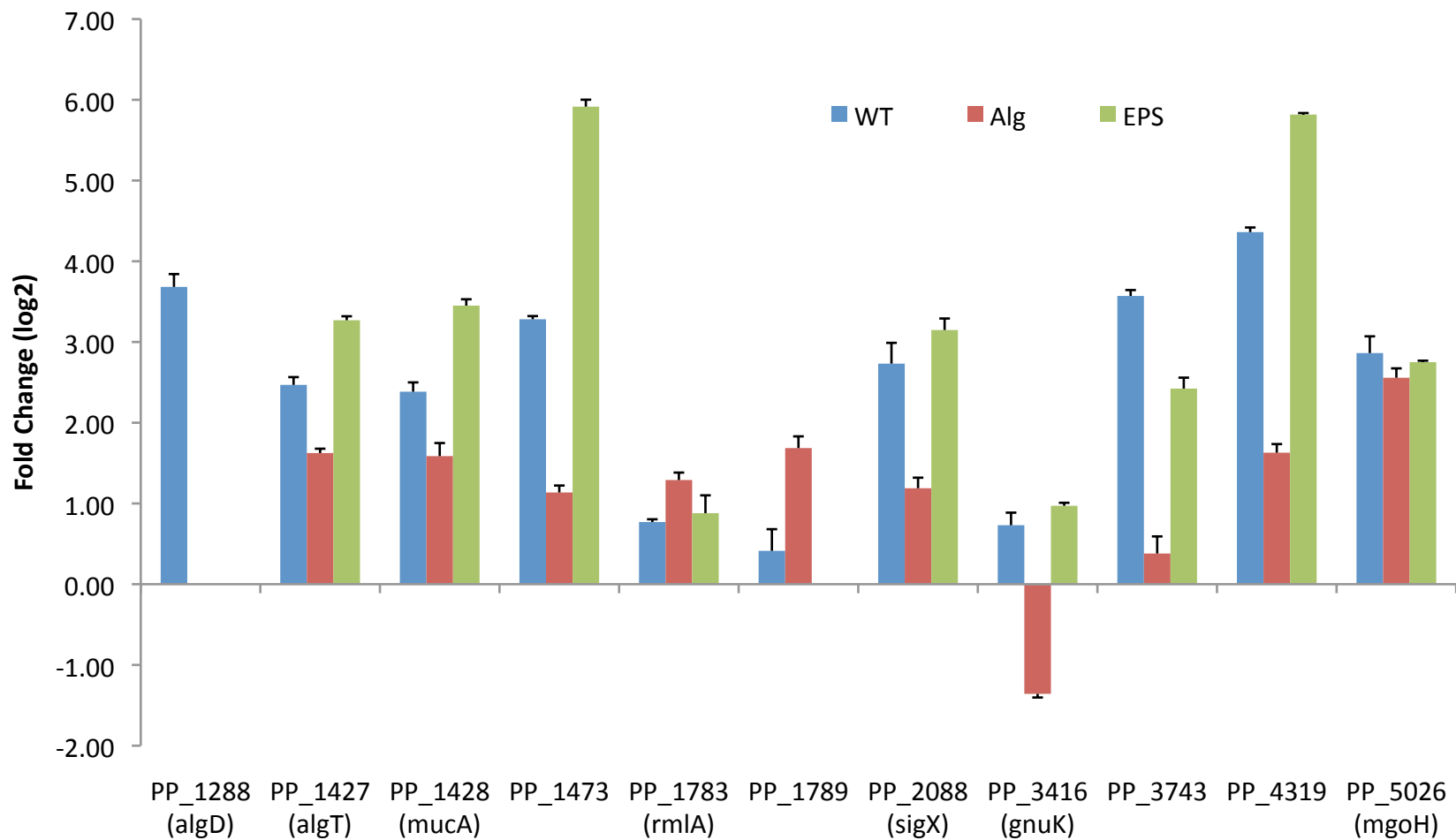

Supplement: Figure S1 — qRT-PCR quantification of the selected transcripts in Pseudomonas putida KT2440 WT and its mutants deficient in exopolysaccharide synthesis (Alg− and EPS−) under water-limited (−0.4 MPa Ψm) relative to water-replete (−0.5 kPa Ψm) conditions. Error bars stand for the standard deviations of the fold-change values. [file mbo30003-0457-sd1.pdf]

a

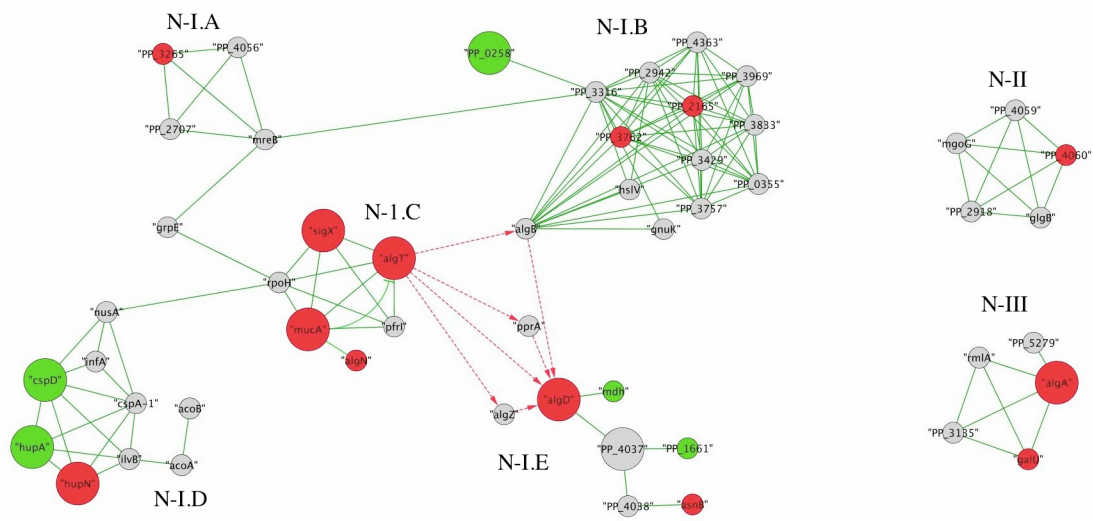

b

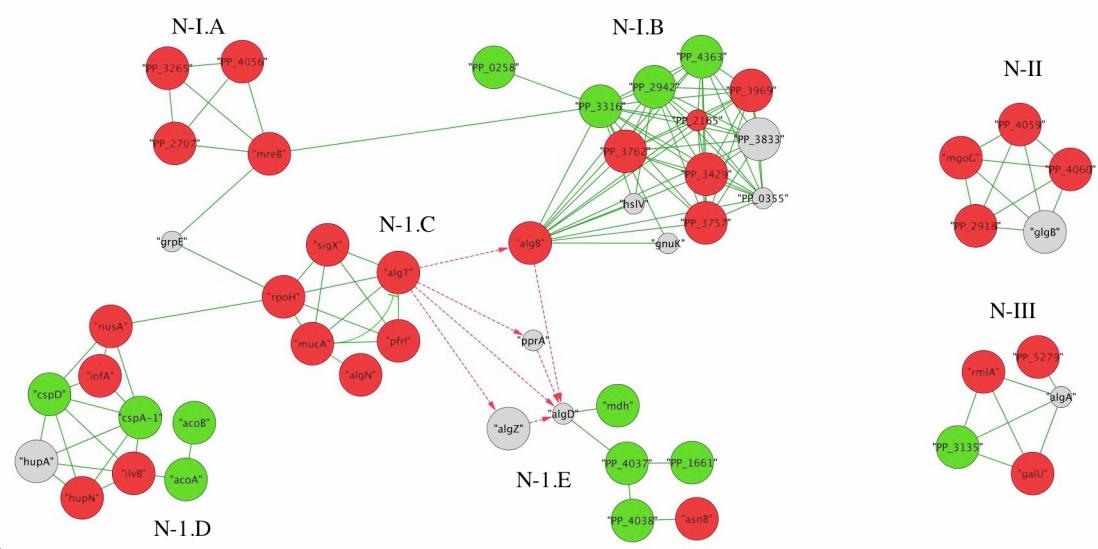

c

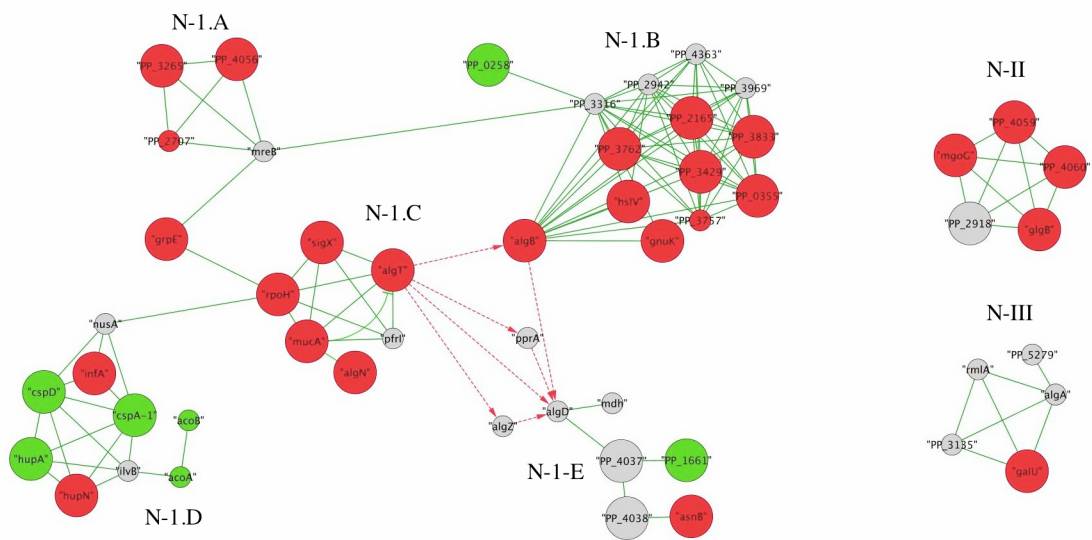

Supplement: Figure S2 — Protein–protein interaction network showing significantly differentially expressed genes in Pseudomonas putida KT2440 strains under water-limited (−0.4 MPa Ψm) relative to water-replete (−0.5 kPa Ψm) condition: (A) WT, (B) alginate mutant, (C) EPS mutant. Node size corresponds to statistical significance based on FDR where a bigger node corresponds to higher FDR. Red, green, and gray nodes correspond to upregulated, downregulated, and nonsignificant genes, respectively. Edge colors correspond to the interactions retrieved by Park et al. (2009) as green and by Wozniak and Ohman (1994) and Remminghorst and Rehm (2006) as dotted red. [file mbo30003-0457-sd2.pdf]
