# Supplementary material for: Colony morphology and transcriptome profiling of Pseudomonas putida KT2440 and its mutants deficient in alginate or all EPS synthesis under controlled matric potentials
Source: Microbiologyopen. 2014 Jun 10;3(4):457–69. doi: 10.1002/mbo3.180 (PMC4287175; doi:10.1002/mbo3.180)
Supplement: Table S4 — Primers used in the qRT-PCR [file mbo30003-0457-sd6.docx]

| **ID** | **Primer** | **Sequence** | **Reference** |
| --- | --- | --- | --- |
| PP_1288 | AlgD-RT2F | 5´- CCACCCGCCGTCACACCATC -3´ | This study |
| PP_1288 | AlgD-RT2R | 5´- TTGACCGCCACGCCGAAGTC -3´ | This study |
| PP_1427 | AlgT-RT2F | 5´-ACGGGCTGAGTTACGAAGACATTG-3´ | Li et al. (2010) |
| PP_1427 | AlgT-RT2R | 5´-AACGGCTGCAGGGCTTTATCTATG-3´ | Li et al. (2010) |
| PP_1428 | MucA-RT2F | 5´-TGAAGCTTTGCAGGAATCGCTGTC-3´ | Li et al. (2010) |
| PP_1428 | MucA-RT2R | 5´-TTAGGCAGCAGCAGTTCCTTGTG-3´ | Li et al. (2010) |
| PP_1463 | RimM-RT2F | 5´-GAGCGGTTACGAAATCTGCATCCC-3´ | Li et al. (2010) |
| PP_1463 | RimM-RT2R | 5´-CATTACATCGTTCGCACCGGTCTC-3´ | Li et al. (2010) |
| PP_1473 |  | 5´- AAGAATCTGCAGCCTGCTCAA -3´ | This study |
| PP_1473 |  | 5´- AGGGTCGTGAACGGTCAGTT -3´ | This study |
| PP_1783 | RmlA-RT2F | 5´- CCTGTCATACGCAATACAACCAA -3´ | This study |
| PP_1783 | RmlA-RT2R | 5´- ACCGAGAACCAACGCAGAAG -3´ | This study |
| PP_1789 |  | 5´- CGCTATGCTGAAAACGTTAGCA -3´ | This study |
| PP_1789 |  | 5´- CGATCAACCAGCGAGTATTCG -3´ | This study |
| PP_2088 | SigX-RT2F | 5´- GAAAGTGCTGTACGGGCTGAA -3´ | This study |
| PP_2088 | SigX-RT2R | 5´- CTTGCGGTACTGGGTAATGCA -3´ | This study |
| PP_3416 | GnuK-RT2F | 5´- AAAACATCCGCAAGATGAGC -3´ | Wang and Nomura (2010) |
| PP_3416 | GnuK-RT2R | 5´- GTTCGAGGAACACGAACACC -3´ | Wang and Nomura (2010) |
| PP_3743 |  | 5´- TTCGCTGTATTCGACCTTCAAG -3´ | This study |
| PP_3743 |  | 5´- GCGCCGACTCCACATAGG -3´ | This study |
| PP_4319 |  | 5´- GGAGAAGCAGCCCAATACCTT-3´ | This study |
| PP_4319 |  | 5´- GTACACCACTGCTGTCAGTTTCCT -3´ | This study |
| PP_5026 | MgoG-RT2F | 5´- CAAGGACAAGACCCCGTTCA-3´ | This study |
| PP_5026 | MgoG-RT2R | 5´- GTACTTGATTTCCTCGACCTTGGT -3´ | This study |

**References**

1. Wang, Q., and Nomura, C.T. (2010) Monitoring differences in gene expression levels and polyhydroxyalkanoate (PHA) production in Pseudomonas putida KT2440 grown on different carbon sources. J.Biosci. BioEng. 110(6): 653-659
2. Li, X., Nielsen, L., Nolan, C., and Halverson, L.J. (2010) Transient alginate gene expression by Pseudomonas putida biofilm residents under water-limiting conditions reflects adaptation to the local environment. Environ. Microbiol. 12:1578-1590
