# Supplementary material for: Colony morphology and transcriptome profiling of Pseudomonas putida KT2440 and its mutants deficient in alginate or all EPS synthesis under controlled matric potentials
Source: Microbiologyopen. 2014 Jun 10;3(4):457–69. doi: 10.1002/mbo3.180 (PMC4287175; doi:10.1002/mbo3.180)
Supplement: Supplementary file 7 [file mbo30003-0457-sd7.docx]

**Supplementary Tables**

Table S1 Log_2_-fold changes for significantly differentially expressed genes when comparing the *P. putida* KT2440 EPS deficient mutant cells to the WT cells or to each other under water limited (-0.4 MPa Ψ_m_) and water replete (-0.5 kPa Ψ_m_) condition(Comparison-I)

Table S2 Log_2_-fold changes for significantly differentially expressed genes listed according to their role categories in *P. putida* KT2440 WT and its mutants deficient in EPS synthesis when comparing cells grown underwater limited (-0.4 MPa Ψ_m_) relative to water replete (-0.5 kPa Ψ_m_) condition (Comparison II).

Table S3 Confidence scores of the interactions in the protein-protein interaction network of *P. putida* KT2440 acquired by Park *et al.* (2009)

Table S4 Primers used in the qRT-PCR

**Supplementary Figures**

Figure S1. qRT-PCR quantification of the selected transcripts in *P. putida*  KT2440 WT and its mutants deficient in exopolysaccharide synthesis (Alg^-^ and EPS^-^) under water limited (-0.4 MPa Ψ_m_) relative to water replete (-0.5 kPa Ψ_m_) condition . Error bars stand for the standard deviations of the fold change values.

Figure S2. Protein-protein interaction network showing significantly differentially expressed genes in *P. putida* KT2440 strains under water limited (-0.4 MPa Ψ_m_) relative to water replete (-0.5 kPa Ψ_m_) condition: a) wild type b) alginate mutant c) EPS mutant. Node size corresponds to statistical significance based on FDR where a bigger node corresponds to higher FDR. Red, green, and gray nodes correspond to up-regulated, down-regulated and non-significant genes, respectively. Edge colors correspond to the interactions retrieved by Park *et al.,* (2009) as green and by Wozniak and Ohman (1994) and Remminhorst and Rehm (2006) as dotted red.
